# 1,4,5,8-Tetra-*n*-butyl­anthracene

**DOI:** 10.1107/S1600536810035877

**Published:** 2010-09-15

**Authors:** Chitoshi Kitamura, Hideki Tsukuda, Takeshi Kawase, Takashi Kobayashi, Hiroyoshi Naito

**Affiliations:** a Department of Materials Science and Chemistry, Graduate School of Engineering, University of Hyogo, 2167 Shosha, Himeji, Hyogo 671-2280, Japan; b Department of Physics and Electronics, Graduate School of Engineering, Osaka Prefecture University, 1-1 Gakuencho, Naka-ku, Sakai, Osaka 599-8531, Japan

## Abstract

The mol­ecule of the title compound, C_30_H_42_, occupies a special position on an inversion center. The four butyl side chains have all-*trans* planar conformations, and the alkyl planes are nearly orthogonal to the anthracene plane [C—C—C—C torsion angles of 79.6 (2) and 78.2 (2)°]. The overall mol­ecule has a stair-like shape with the *n*-butyl groups at the 1 and 8 positions extending towards the same side of the anthracene plane. In the crystal structure, mol­ecules adopt a slipped–parallel arrangement without π–π stacking.

## Related literature

For background to solid-state packing effects in electronic and photonic materials, see: Curtis *et al.* (2004 ▶). For the correlation between π–π stacking and fluorescence quantum yields, see: Yoshida *et al.* (2002 ▶). For related structures and their solid-state fluorescence, see: Kitamura, Abe *et al.* (2007 ▶); Kitamura, Ohara *et al.* (2007 ▶); Kitamura *et al.* (2010 ▶).
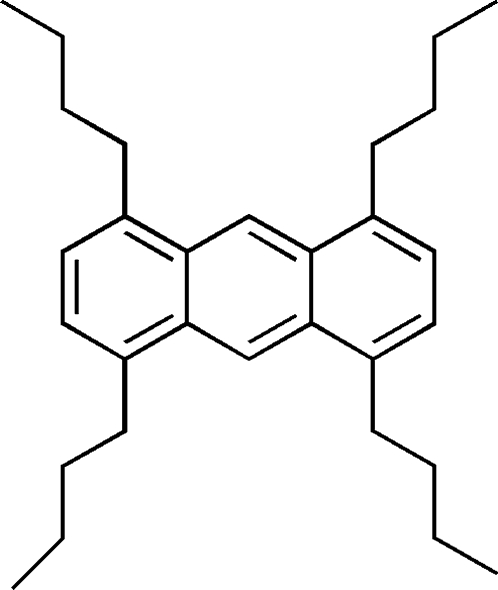


## Experimental

### 

#### Crystal data

C_30_H_42_
*M* *_r_* = 402.64
Triclinic, 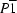

*a* = 4.793 (2) Å
*b* = 11.497 (6) Å
*c* = 11.753 (6) Å
α = 83.052 (14)°
β = 82.205 (15)°
γ = 83.202 (15)°
*V* = 633.5 (5) Å^3^
*Z* = 1
Mo *K*α radiation
μ = 0.06 mm^−1^
*T* = 223 K
0.50 × 0.05 × 0.02 mm

#### Data collection

Rigaku/MSC Mercury CCD area-detector diffractometer
Absorption correction: numerical (*NUMABS*; Higashi, 2000 ▶) *T*_min_ = 0.988, *T*_max_ = 0.999
5371 measured reflections
3103 independent reflections
1361 reflections with *I* > 2σ(*I*)
*R*_int_ = 0.033

#### Refinement

*R*[*F* ^2^ > 2σ(*F* ^2^)] = 0.073
*wR*(*F* ^2^) = 0.246
*S* = 0.98
3103 reflections
138 parameters
H-atom parameters constrained
Δρ_max_ = 0.31 e Å^−3^
Δρ_min_ = −0.17 e Å^−3^

### 

Data collection: *CrystalClear* (Rigaku/MSC, 2006 ▶); cell refinement: *CrystalClear*; data reduction: *CrystalClear*; program(s) used to solve structure: *SIR2004* (Burla *et al.*, 2005 ▶); program(s) used to refine structure: *SHELXL97* (Sheldrick, 2008 ▶); molecular graphics: *ORTEP-3 for Windows* (Farrugia, 1997 ▶); software used to prepare material for publication: *WinGX* (Farrugia, 1999 ▶).

## Supplementary Material

Crystal structure: contains datablocks global, I. DOI: 10.1107/S1600536810035877/ya2127sup1.cif

Structure factors: contains datablocks I. DOI: 10.1107/S1600536810035877/ya2127Isup2.hkl

Additional supplementary materials:  crystallographic information; 3D view; checkCIF report

## Figures and Tables

**Fig. 1.**
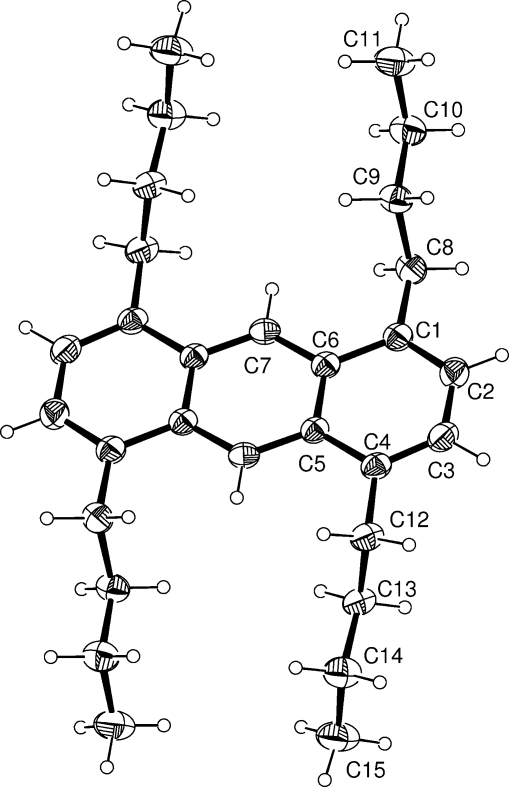
Molecular structure of the title compound; displacement ellipsoids are drawn at the 30° probability level; the unlabelled atoms are derived by the symmetry transformation -x + 2, -y + 1, -z + 1.

**Fig. 2.**
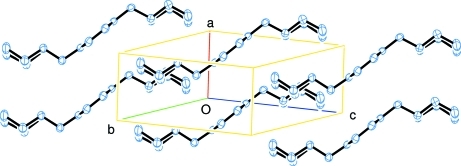
The packing diagram of the title compound viewed down the long molecular axis of anthracene ring; hydrogen atoms are omitted for clarity.

## supplementary crystallographic information

### Comment

Solid-state packing effects play an important role in the performance of electronic and photonic materials (Curtis *et al.*, 2004). However, there has been relatively little research on the correlation between solid-state packing patterns and fluorescence properties. Therefore, molecular design principles which could allow to control solid-state fluorescence are not fully understood.

We have recently found that the introduction of different alkyl side chains onto anthracene nucleus at the 1, 4, 5, and 8 positions brought about considerable variety in alkyl conformations, packing patterns, and solid-state fluorescence properties. As a part of systematic investigation of this phenomena, we report herein the structure of the title compound, namely, the anthracene tetra-*n*-butyl derivative (Fig.1).

The molecule occupies a special position in the inversion center. The bond lengths and bond angles are comparable to those of other 1,4,5,8-tetraalkylanthracenes (Kitamura, Abe *et al.*, 2007). Four butyl side chains adopt all-*trans* planar conformations, and the alkyl planes are nearly orthogonal to the anthracene plane; the torsion angles of C6—C1—C8—C9 and C5—C4—C12—C13 are 79.6 (2) and 78.2 (2)°, respectively. Thus, the molecule of the title compound has a stair-like shape, and two butyl groups at the 1 and 8 positions extend towards the same side of the anthracene plane. This molecular structure is similar to that of 1,4,7,10-tetra-n-butyltetracene (Kitamura, Ohara *et al.*, 2007; Kitamura *et al.*, 2010).

In crystal the molecules show a slipped-parallel arrangement without π-π stacking (Fig. 2). Such packing, as has been shown (Yoshida *et al.*, 2002), can enhance fluorescence quantum yields because of the same directions of transition dipole moments for all molecules as well as no concentration quenching (Kitamura, Abe *et al.*, 2007).

To examine the influence of crystal packing on the solid-state fluorescence properties, the fluorescence spectrum and the absolute quantum yield of the title compound were measured with a Hamamatsu Photonics PMA11 calibrated optical multichannel analyzer with a solid-state blue laser (*λ*_ex_ = 377 nm) and a Labsphere IS-040-SF integrating sphere. The crystals exhibited a broad fluorescence spectrum with a fluorescence maximum at 450 nm, a shoulder peak around 467 nm and very high quantum yield (*Φ* = 0.78). Among 1,4,5,8-tetraalkylanthracenes, only the propyl derivative had higher quantum yield of 0.85 (Kitamura, Abe *et al.*, 2007), which is of course still quite comparable to that of the title compound.

### Experimental

1,4,5,8-Tetra-n-butylanthracene was prepared according to the method described by Kitamura, Abe *et al.* (2007). A mixture of 2,5-dibutylfuran (2.99 g, 16.3 mmol) and 1,2,4,5-tetrabromobenzene (3.10 g, 7.88 mmol) in dry toluene (50 ml) was cooled to 243 K. To the mixture, 1.6 *M n*-BuLi in hexane (14.8 ml, 23.7 mmol) was added dropwise over 15 min. Then the mixture was warmed up to room temperature over 2 h and stirred at room temperature for additional 18 h. After quenching with water, the aqueous layer was extracted with CHCl_3_. The combined organic layer was washed with brine and dried over Na_2_SO_4_. After evaporation, the residue was subjected to slica-gel chromatography with (2:1)-hexane/CHCl_3_ to afford bis(furan)adduct as an orange solid (602 mg, 18°). The bis(furan)adduct (602 mg, 1.38 mmol) in EtOH (60 ml) was hydrogenated over 10° Pd/C (125 mg) under atmospheric pressure at room temperature for 3 h. The catalyst was removed by filtration, and the filtrate was evaporated under reduced pressure. To the residue, an ice-cooled solution of (1:5)-conc. HCl/Ac_2_O (6 ml) was added. The mixture was stirred at room temperature for 3 h. After cooling with ice, water was added into the mixture. The resultant mixture was extracted with CHCl_3_, and the extract was washed with aqueous Na_2_CO_3_ and brine, and dried over Na_2_SO_4_. After evaporation of the solvent, column chromatography on silica gel with (2:1)-hexane/CHCl_3_ gave the title compound as a pale yellow solid (412 mg, 45°). Recrystallization was performed with hexane to obtain colorless single crystals of the title compound. ^1^H-NMR: *δ* 1.00 (t, J = 7.2 Hz, 12H), 1.48–1.55 (m, 8H), 1.80–1.86 (m, 8H), 3.18 (t, J = 7.6 Hz, 8H), 7.22 (s, 4H), 8.80 (s, 2H); ^13^C-NMR: *δ* 14.06, 23.05, 33.03, 33.30, 120.04, 124.66, 130.03, 137.03; EIMS: *m*/*z* (°) 402 (100); Elemental analysis for C_30_H_42_: C, 89.49; H, 10.51. Found: C, 89.41; H, 10.60.

### Refinement

All the H atoms were positioned geometrically and refined using a riding model with C—H bonds of 0.94 \%A, 0.98\%A, and 0.97\%A for aromatic, methylene, and methyl groups, respectively, and *U*_iso_(H) = 1.2*U*_eq_(C) [*U*_iso_(H) = 1.5*U*_eq_(C) for methyl H atoms].

### Crystal data

**Table:**

C_30_H_42_	*Z* = 1
*M**_r_* = 402.64	*F*(000) = 222
Triclinic, *P*1	*D*_x_ = 1.055 Mg m^−^^3^
Hall symbol: -P 1	Melting point: 368 K
*a* = 4.793 (2) Å	Mo *K*α radiation, λ = 0.71073 Å
*b* = 11.497 (6) Å	Cell parameters from 1148 reflections
*c* = 11.753 (6) Å	θ = 2.4–30.0°
α = 83.052 (14)°	µ = 0.06 mm^−^^1^
β = 82.205 (15)°	*T* = 223 K
γ = 83.202 (15)°	Needle, colorless
*V* = 633.5 (5) Å^3^	0.50 × 0.05 × 0.02 mm

### Data collection

**Table:**

Rigaku/MSC Mercury CCD area-detector diffractometer	3103 independent reflections
Radiation source: rotating-anode X-ray tube	1361 reflections with *I* > 2σ(*I*)
graphite	*R*_int_ = 0.033
Detector resolution: 14.7059 pixels mm^-1^	θ_max_ = 28.3°, θ_min_ = 2.6°
φ and ω scans	*h* = −6→5
Absorption correction: numerical (*NUMABS*; Higashi, 2000)	*k* = −12→15
*T*_min_ = 0.988, *T*_max_ = 0.999	*l* = −10→15
5371 measured reflections	

### Refinement

**Table:**

Refinement on *F*^2^	Primary atom site location: structure-invariant direct methods
Least-squares matrix: full	Hydrogen site location: inferred from neighbouring sites
*R*[*F*^2^ > 2σ(*F*^2^)] = 0.073	H-atom parameters constrained
*wR*(*F*^2^) = 0.246	*w* = 1/[σ^2^(*F*_o_^2^) + (0.1174*P*)^2^] where *P* = (*F*_o_^2^ + 2*F*_c_^2^)/3
*S* = 0.98	(Δ/σ)_max_ < 0.001
3103 reflections	Δρ_max_ = 0.31 e Å^−^^3^
138 parameters	Δρ_min_ = −0.17 e Å^−^^3^
0 restraints	

### Special details

**Table:**

Geometry. All s.u.'s (except the s.u. in the dihedral angle between two l.s. planes) are estimated using the full covariance matrix. The cell s.u.'s are taken into account individually in the estimation of s.u.'s in distances, angles and torsion angles; correlations between s.u.'s in cell parameters are only used when they are defined by crystal symmetry. An approximate (isotropic) treatment of cell s.u.'s is used for estimating s.u.'s involving l.s. planes.
Refinement. Refinement of *F*^2^ against ALL reflections. The weighted *R*-factor *wR* and goodness of fit *S* are based on *F*^2^, conventional *R*-factors *R* are based on *F*, with *F* set to zero for negative *F*^2^. The threshold expression of *F*^2^ > 2σ(*F*^2^) is used only for calculating *R*-factors(gt) *etc*. and is not relevant to the choice of reflections for refinement. *R*-factors based on *F*^2^ are statistically about twice as large as those based on *F*, and *R*- factors based on ALL data will be even larger.

### Fractional atomic coordinates and isotropic or equivalent isotropic displacement parameters (Å^2^)

**Table:**

	*x*	*y*	*z*	*U*_iso_*/*U*_eq_	
C1	0.7400 (4)	0.4153 (2)	0.3252 (2)	0.0481 (6)	
C2	0.8025 (5)	0.2989 (2)	0.3077 (2)	0.0584 (7)	
H2	0.7214	0.2695	0.25	0.07*	
C3	0.9869 (5)	0.2214 (2)	0.3745 (2)	0.0583 (7)	
H3	1.0259	0.1424	0.3588	0.07*	
C4	1.1078 (4)	0.2577 (2)	0.4598 (2)	0.0481 (6)	
C5	1.0540 (4)	0.37919 (19)	0.48075 (18)	0.0423 (6)	
C6	0.8697 (4)	0.45829 (19)	0.41302 (18)	0.0430 (6)	
C7	0.8223 (4)	0.5758 (2)	0.43552 (18)	0.0461 (6)	
H7	0.7003	0.6274	0.3918	0.055*	
C8	0.5481 (4)	0.4958 (2)	0.2522 (2)	0.0544 (7)	
H8A	0.424	0.5482	0.3016	0.065*	
H8B	0.428	0.4482	0.2198	0.065*	
C9	0.7059 (4)	0.5708 (2)	0.1533 (2)	0.0560 (7)	
H9A	0.8283	0.6173	0.1858	0.067*	
H9B	0.8281	0.5182	0.1035	0.067*	
C10	0.5175 (5)	0.6530 (2)	0.0806 (2)	0.0684 (8)	
H10A	0.3991	0.7072	0.1298	0.082*	
H10B	0.3916	0.6069	0.0495	0.082*	
C11	0.6765 (7)	0.7242 (3)	−0.0187 (3)	0.0873 (10)	
H11A	0.8021	0.7701	0.0111	0.131*	
H11B	0.5427	0.7767	−0.0606	0.131*	
H11C	0.7867	0.6714	−0.0703	0.131*	
C12	1.2822 (5)	0.1713 (2)	0.5349 (2)	0.0563 (7)	
H12A	1.4472	0.2077	0.5493	0.068*	
H12B	1.3514	0.1022	0.4939	0.068*	
C13	1.1155 (5)	0.1314 (2)	0.6507 (2)	0.0563 (7)	
H13A	1.0287	0.2014	0.6872	0.068*	
H13B	0.9622	0.0881	0.636	0.068*	
C14	1.2883 (5)	0.0551 (2)	0.7335 (2)	0.0708 (8)	
H14A	1.4368	0.0995	0.751	0.085*	
H14B	1.3813	−0.0135	0.6961	0.085*	
C15	1.1173 (6)	0.0126 (3)	0.8457 (2)	0.0865 (10)	
H15A	1.0222	0.0798	0.8828	0.13*	
H15B	1.2427	−0.0335	0.8963	0.13*	
H15C	0.9777	−0.0359	0.8297	0.13*	

### Atomic displacement parameters (Å^2^)

**Table:**

	*U*^11^	*U*^22^	*U*^33^	*U*^12^	*U*^13^	*U*^23^
C1	0.0437 (10)	0.0523 (15)	0.0476 (13)	−0.0138 (10)	−0.0001 (9)	0.0003 (11)
C2	0.0682 (14)	0.0565 (17)	0.0521 (15)	−0.0177 (12)	−0.0026 (12)	−0.0069 (13)
C3	0.0745 (14)	0.0430 (14)	0.0546 (15)	−0.0093 (11)	0.0047 (12)	−0.0055 (12)
C4	0.0515 (11)	0.0456 (14)	0.0438 (13)	−0.0073 (10)	0.0046 (10)	−0.0005 (11)
C5	0.0422 (10)	0.0419 (13)	0.0403 (12)	−0.0073 (9)	0.0048 (9)	−0.0025 (10)
C6	0.0393 (10)	0.0465 (14)	0.0409 (12)	−0.0092 (9)	0.0042 (9)	−0.0014 (11)
C7	0.0430 (10)	0.0493 (15)	0.0426 (12)	−0.0051 (9)	0.0012 (9)	0.0022 (10)
C8	0.0469 (11)	0.0663 (17)	0.0508 (14)	−0.0147 (11)	−0.0041 (10)	−0.0035 (12)
C9	0.0501 (11)	0.0670 (17)	0.0487 (14)	−0.0074 (11)	−0.0058 (10)	0.0034 (12)
C10	0.0667 (14)	0.073 (2)	0.0632 (17)	−0.0037 (13)	−0.0133 (13)	0.0043 (15)
C11	0.105 (2)	0.085 (2)	0.070 (2)	−0.0111 (17)	−0.0247 (17)	0.0186 (17)
C12	0.0641 (13)	0.0464 (15)	0.0532 (15)	−0.0004 (11)	0.0033 (11)	−0.0006 (12)
C13	0.0617 (12)	0.0459 (15)	0.0572 (15)	−0.0038 (10)	−0.0026 (11)	0.0041 (12)
C14	0.0745 (15)	0.070 (2)	0.0613 (17)	0.0007 (13)	−0.0017 (13)	0.0026 (15)
C15	0.108 (2)	0.085 (2)	0.0570 (18)	0.0006 (17)	−0.0018 (16)	0.0125 (16)

### Geometric parameters (Å, °)

**Table:**

C1—C2	1.370 (3)	C9—H9B	0.98
C1—C6	1.438 (3)	C10—C11	1.513 (4)
C1—C8	1.503 (3)	C10—H10A	0.98
C2—C3	1.422 (3)	C10—H10B	0.98
C2—H2	0.94	C11—H11A	0.97
C3—C4	1.353 (3)	C11—H11B	0.97
C3—H3	0.94	C11—H11C	0.97
C4—C5	1.435 (3)	C12—C13	1.530 (3)
C4—C12	1.502 (3)	C12—H12A	0.98
C5—C7^i^	1.394 (3)	C12—H12B	0.98
C5—C6	1.438 (3)	C13—C14	1.498 (3)
C6—C7	1.394 (3)	C13—H13A	0.98
C7—C5^i^	1.394 (3)	C13—H13B	0.98
C7—H7	0.94	C14—C15	1.515 (3)
C8—C9	1.530 (3)	C14—H14A	0.98
C8—H8A	0.98	C14—H14B	0.98
C8—H8B	0.98	C15—H15A	0.97
C9—C10	1.500 (3)	C15—H15B	0.97
C9—H9A	0.98	C15—H15C	0.97
			
C2—C1—C6	117.9 (2)	C11—C10—H10A	108.8
C2—C1—C8	120.7 (2)	C9—C10—H10B	108.8
C6—C1—C8	121.3 (2)	C11—C10—H10B	108.8
C1—C2—C3	121.8 (2)	H10A—C10—H10B	107.7
C1—C2—H2	119.1	C10—C11—H11A	109.5
C3—C2—H2	119.1	C10—C11—H11B	109.5
C4—C3—C2	122.1 (2)	H11A—C11—H11B	109.5
C4—C3—H3	118.9	C10—C11—H11C	109.5
C2—C3—H3	118.9	H11A—C11—H11C	109.5
C3—C4—C5	118.6 (2)	H11B—C11—H11C	109.5
C3—C4—C12	120.7 (2)	C4—C12—C13	112.63 (17)
C5—C4—C12	120.7 (2)	C4—C12—H12A	109.1
C7^i^—C5—C4	122.1 (2)	C13—C12—H12A	109.1
C7^i^—C5—C6	118.3 (2)	C4—C12—H12B	109.1
C4—C5—C6	119.6 (2)	C13—C12—H12B	109.1
C7—C6—C5	118.1 (2)	H12A—C12—H12B	107.8
C7—C6—C1	122.0 (2)	C14—C13—C12	114.55 (19)
C5—C6—C1	119.9 (2)	C14—C13—H13A	108.6
C6—C7—C5^i^	123.6 (2)	C12—C13—H13A	108.6
C6—C7—H7	118.2	C14—C13—H13B	108.6
C5^i^—C7—H7	118.2	C12—C13—H13B	108.6
C1—C8—C9	113.77 (16)	H13A—C13—H13B	107.6
C1—C8—H8A	108.8	C13—C14—C15	113.7 (2)
C9—C8—H8A	108.8	C13—C14—H14A	108.8
C1—C8—H8B	108.8	C15—C14—H14A	108.8
C9—C8—H8B	108.8	C13—C14—H14B	108.8
H8A—C8—H8B	107.7	C15—C14—H14B	108.8
C10—C9—C8	114.48 (17)	H14A—C14—H14B	107.7
C10—C9—H9A	108.6	C14—C15—H15A	109.5
C8—C9—H9A	108.6	C14—C15—H15B	109.5
C10—C9—H9B	108.6	H15A—C15—H15B	109.5
C8—C9—H9B	108.6	C14—C15—H15C	109.5
H9A—C9—H9B	107.6	H15A—C15—H15C	109.5
C9—C10—C11	113.8 (2)	H15B—C15—H15C	109.5
C9—C10—H10A	108.8		
			
C6—C1—C2—C3	0.7 (3)	C8—C1—C6—C7	0.6 (3)
C8—C1—C2—C3	179.03 (17)	C2—C1—C6—C5	−1.2 (3)
C1—C2—C3—C4	0.9 (3)	C8—C1—C6—C5	−179.49 (16)
C2—C3—C4—C5	−1.9 (3)	C5—C6—C7—C5^i^	0.6 (3)
C2—C3—C4—C12	174.95 (18)	C1—C6—C7—C5^i^	−179.46 (16)
C3—C4—C5—C7^i^	−177.93 (17)	C2—C1—C8—C9	−98.6 (2)
C12—C4—C5—C7^i^	5.2 (3)	C6—C1—C8—C9	79.6 (2)
C3—C4—C5—C6	1.3 (3)	C1—C8—C9—C10	−179.2 (2)
C12—C4—C5—C6	−175.48 (16)	C8—C9—C10—C11	−178.5 (2)
C7^i^—C5—C6—C7	−0.6 (3)	C3—C4—C12—C13	−98.5 (2)
C4—C5—C6—C7	−179.91 (16)	C5—C4—C12—C13	78.2 (3)
C7^i^—C5—C6—C1	179.49 (16)	C4—C12—C13—C14	−173.9 (2)
C4—C5—C6—C1	0.2 (3)	C12—C13—C14—C15	−177.7 (2)
C2—C1—C6—C7	178.90 (17)		

Symmetry codes: (i) −*x*+2, −*y*+1, −*z*+1.

## References

[bb1] Burla, M. C., Caliandro, R., Camalli, M., Carrozzini, B., Cascarano, G. L., De Caro, L., Giacovazzo, C., Polidori, G. & Spagna, R. (2005). *J. Appl. Cryst.* **38**, 381–388.

[bb2] Curtis, M. D., Cao, J. & Kampf, J. W. (2004). *J. Am. Chem. Soc.* **126**, 4318–4328. 10.1021/ja0397916 15053622

[bb3] Farrugia, L. J. (1997). *J. Appl. Cryst.* **30**, 565.

[bb4] Farrugia, L. J. (1999). *J. Appl. Cryst.* **32**, 837–838.

[bb5] Higashi, T. (2000). *NUMABS.* Rigaku Corporation, Tokyo, Japan.

[bb6] Kitamura, C., Abe, Y., Kawatsuki, N., Yoneda, A., Asada, K., Kobayashi, A. & Naito, H. (2007). *Mol. Cryst. Liq. Cryst.* **474**, 119–135.

[bb7] Kitamura, C., Abe, Y., Ohara, T., Yoneda, A., Kawase, T., Kobayashi, A., Naito, H. & Komatsu, T. (2010). *Chem. Eur. J.* **16**, 890–898. 10.1002/chem.200901668 19937617

[bb8] Kitamura, C., Ohara, T., Kawatsuki, N., Yoneda, A., Kobayashi, A., Naito, H., Komatsu, T. & Kitamura, T. (2007). *CrystEngComm*, **9**, 644–647.

[bb9] Rigaku/MSC (2006). *CrystalClear.* Rigaku Corporation, Tokyo, Japan.

[bb10] Sheldrick, G. M. (2008). *Acta Cryst.* A**64**, 112–122. 10.1107/S0108767307043930 18156677

[bb11] Yoshida, K., Ooyama, Y., Miyazaki, H. & Watanabe, S. (2002). *J. Chem. Soc. Perkin Trans. 2*, pp. 700–707.

